# Support for mothers and their families after life-threatening illness in pregnancy and childbirth: a qualitative study in primary care

**DOI:** 10.3399/bjgp15X686461

**Published:** 2015-09-01

**Authors:** Lisa Hinton, Louise Locock, Marian Knight

**Affiliations:** Health Experiences Research Group, Nuffield Department of Primary Care Health Sciences, Medical Sciences Division, University of Oxford, Oxford.; Health Experiences Research Group, Nuffield Department of Primary Care Health Sciences, Medical Sciences Division, University of Oxford, Oxford and NIHR Oxford Biomedical Research Centre, Oxford.; National Perinatal Epidemiology Unit, Nuffield Department of Population Health, University of Oxford, Oxford.

**Keywords:** childbirth, maternal illness, primary care, qualitative, support

## Abstract

**Background:**

One in 100 women who give birth in the UK develop life-threatening illnesses during childbirth. Without urgent medical attention these illnesses could lead to the mother’s death. Little is known about how the experience of severe illness in childbirth affects the mother, baby, and family.

**Aim:**

As part of the UK National Maternal Near-miss Surveillance Programme, this study explored the experiences of women and their partners of life-threatening illnesses in childbirth, to identify the long-term impact on women and their families.

**Design and setting:**

Qualitative study based on semi-structured narrative interviews. Interviews were conducted in patients’ homes in England and Scotland from 2010 to 2014.

**Method:**

An in-depth interview study was conducted with 36 women and 11 partners. A maximum variation sample was sought and interviews transcribed for thematic analysis with constant comparison.

**Results:**

Women’s birth-related illnesses often had long-lasting effects on their mental as well as physical health, including anxiety, panic attacks, and post-traumatic stress disorder. In some cases the partner’s mental health was also affected. Women often described feeling isolated. Their experiences can have a profound impact on their relationships, family life, career, and future fertility. While some women described receiving good support from their GP, others felt there was little support available for them or their families after discharge from hospital.

**Conclusion:**

A near-miss event can have long-lasting and major effects on women and their families. Support in primary care, including watchful waiting for mental health impacts, can play a valuable role in helping these families come to terms with their emergency experience. The findings highlight the importance of communication between primary and secondary care.

## INTRODUCTION

It is estimated that for every 100 women giving birth in the UK, there will be one woman experiencing a near miss: an obstetric complication requiring urgent live-saving medical care.^1^ Potentially 8000 women and their families each year have to cope with a life-threatening emergency and its aftermath. The causes of these near misses are varied but include pre-eclampsia, haemorrhage, thrombosis, and sepsis, and may in some cases require an emergency hysterectomy or preterm delivery. Mother and newborn are often separated because women may have to spend time in intensive care or a high-dependency unit. Their babies may be born prematurely and require neonatal intensive care.

Recent studies draw attention to the potential for long-term psychological and emotional impact on women of maternal morbidities.^2^–^6^ In addition to their physical recovery, women can experience anxiety, isolation, and flashbacks in the aftermath. Birth trauma can have lasting consequences that impact negatively on maternal, infant, and family wellbeing.^7^ Medically-complicated pregnancies can impact negatively on breastfeeding rates.^3^,^8^

Women may be discharged from hospital having had major surgery and emergency treatment or time in intensive care. Some may have lost their baby as a result of their illness. Babies delivered preterm may need to spend long periods in neonatal intensive care. These experiences are a long way away from normal birth and physical and psychological follow-up from hospitals varies around the country.

The aim of this study was to explore the long-term impact of a near-miss obstetric emergency. The study examines specifically the primary care support and services women may need after discharge. It was not possible to find any previous work that has directly addressed this issue. The study draws on qualitative interviews with women and their partners who experienced a near-miss event in childbirth in the UK as part of the National Maternal Near-miss Surveillance Programme.

## METHOD

Women who experienced a life-threatening complication in pregnancy (defined as ‘severe maternal illnesses which, without urgent medical attention, would lead to a mother’s death’)^9^ were invited to take part in an interview study. The women’s partners were also invited.

### Sample

It was aimed to include a maximum variation sample of women living in the UK,^10^,^11^ covering a wide range of conditions, based on the principal causes of direct maternal deaths identified in recent (2011) maternal death enquiry reports (Table 1).^12^ The overall sample included 36 females, and 10 male and one female partner (Table 2).

**Table 1.** Conditions experienced by participants

**Condition^a^**	***n* (Partner, *n*)**
Uterine rupture	4 (2)
Haemorrhage	5 (2)
Haemorrhage and hysterectomy	9 (2)
Placenta praevia	3 (1)
Placenta percreta	2
Placental abruption	1 (1)
Amniotic fluid embolism	3 (2)
Pulmonary embolism	5 (1)
Pre-eclampsia	3
HELLP syndrome	2 (1)
Septicaemia	2
Other, for example appendicitis or failed intubation	4 (1)

a Some women had multiple morbidities and appear in more than one category. HELLP = H (haemolysis) EL (elevated liver enzymes) LP (low platelet count).

**Table 2.** Sociodemographic characteristics of participants (*n* = 47)

**Characteristic**	***n***
**Age at the time of interview, years**	
21–30	3
31–40	31
≥40	13

**Age at time of near miss event, years**	
21–30	11
31–40	31
≥40	5

**Sex**	
Female (mothers)	36
Male or female (partners)	11 (10 male, one female partner)

**Occupation**	
Professional	21
Other non-manual	13
Skilled manual	4
Unskilled manual	2
Other, such as housewife or student	7

**Ethnic group**	
White British	42
British Pakistani	1
White Australian	2
White Israeli	1
British Somali	1

**Time since near miss, years**	
<1	9
1–2	16
2–5	16
5–9	4
≥10	2

**How this fits in?**

One in 100 women who give birth in the UK develop life-threatening illnesses during childbirth. Without urgent medical attention these illnesses could lead to the mother’s death. Research into the long-term impact of these experiences indicates that they have long-lasting effects on women’s mental as well as physical health, and impact their families. Support in primary care can play a valuable role in helping these families come to terms with their emergency experience.

Recruitment packs were distributed through a number of routes to ensure a wide, varied sample; routes included support groups, the National Childbirth Trust, social network forums (Mumsnet and Netmums), a metropolitan newspaper advertisement, intensive care clinicians contacted through the Intensive Care National Audit and Research Centre, advertisement in the UK Obstetric Surveillance System newsletter, and word of mouth. To try and reach a wider ethnic minority population, recruitment packs were translated into Bengali and distributed through a consultant in an east London hospital. A British Somali interviewee was recruited through a local women’s support group.

Not all those who volunteered were interviewed, but the authors sought to ensure that a representative range of conditions and times since the event were included, to understand the longer-term effects of a near-miss event. Although the sample included more interviewees from professional classes than others, it does represent a broad socioeconomic diversity.

### Interviews

Interviews took place between 2010 and 2014. After obtaining informed consent, one of the authors interviewed participants in the setting of their choice (usually their home). Participants were asked about their or their partner’s experiences of pregnancy and life-threatening illness. The interview started with an open-ended narrative section where responders described what had happened, followed by a semi-structured section with prompts to explore any relevant issues that had not already emerged, including their recovery and family life since their near miss. The interviews were all audio or videotaped (to allow for dissemination on the website Healthtalk.org), and transcribed verbatim.

### Analysis

The transcripts were read and re-read, a coding frame was constructed and the data coded. Anticipated and emergent themes were then examined across the whole data set as well as in the context of each person’s interview. A qualitative interpretive approach was taken, combining thematic analysis with constant comparison.^13^,^14^ NVivo (version 9) was used to facilitate the analysis.

The analysis presented here focuses on how responders described their experiences after discharge from hospital and particularly their needs for primary care support.

## RESULTS

The women in the study had spent varying amounts of time in hospital as a consequence of their life-threatening illnesses. In addition to their physical recovery, they described the emotional impact of their emergency and the challenges of settling back to normal life. There was often no follow-up, either physical or psychological, from hospital so GPs were in the front line, caring for and supporting women and their families.

### Isolation

Women described the difficulties they faced settling back into their social relationships with family, friends, and their local community. They often felt isolated; physically isolated because they were not well enough to get out to see people and unable to link with normal post-delivery support networks that other mothers could access. Emotionally, women often found it hard to relate to others who had no idea of the trauma they had experienced and how long recovery might take.

One woman felt that her family and friends quickly forgot what she had been through (please note: the ages of the study participants listed after the quotes reflect their age (years) at time of illness, not at interview):

‘As soon as I came out they didn’t care. That’s when I really needed my friends. That’s when I needed people to be there for me.’

(Anna, 29, in intensive care with septicaemia and needed a hysterectomy)

In particular, women felt isolated from other new mothers because their birth experience had been such a traumatic event, which they did not feel they could share. For first-time mothers this was particularly challenging. They felt excluded from normal support routes such as postnatal groups, or local playgroups:

‘I think one of the hardest things was I felt very alienated from my peers at the time ... And it didn’t take long before I distanced myself quite quickly. I felt very bitter, very uncomfortable being round people who seemingly had it so easy.’

(Cara, 29, hysterectomy with her first child)

‘Well, I would have silenced the group if I had even mentioned any of it. So the kind of things that they were mentioning that were bothering them, I was just thinking, try dying in childbirth, you know, I felt like I was so out of, out of kilter with their experiences. So the things that were bothering me were so extreme that there wasn’t a forum for me to discuss it.’

(Paula, 43, amniotic fluid embolism)

‘It’s a really hard thing to describe, but you almost feel excluded by the fact that you’ve had such a major event that you don’t want to always be coming in and saying, “Well, hey, I had the worst scenario”.’

(Karen, 42, postpartum haemorrhage and hysterectomy)

### Physical recovery

Women’s experiences were very varied, due to the wide range and severity of their illnesses and how long they had been hospitalised. Several had undergone major surgery (for example, a hysterectomy, a much more substantial operation than a caesarean section), and 18 had spent some time in high dependency or critical care. Women were often very physically weak, and felt bed or wheelchair-bound, when discharged. Those who had had abdominal surgery (caesarean or hysterectomy) were still in pain, which took several weeks to subside:

‘Very, very tired. Really, really tired. Tired and sore.’

(Karen, 42, hysterectomy)

Responder (R): ‘I couldn’t move. I couldn’t straighten myself up. It was an effort to go upstairs to the toilet. I couldn’t cook. I couldn’t hold the babies. My son was only four and then obviously the middle one now, he was only 11 month old. He wasn’t walking. I couldn’t pick him up and it was like that for at least a week. I was basically just stuck on the couch.’

Interviewer: ‘Okay and did you recover physically quite quickly?’

R: *‘* [um] *No, because I was going to the hospital twice a day to see the baby and spending a lot of hours there.’ * (Kerry, 25, placenta praevia)

A common frustration for women was not being able to look after their children:

*‘I was on a zimmer frame to start off with, to keep my balance and ... and then I had crutches to go home with.* [um] *And that was a whole new challenge, because it was, it was relief to be around my children, but being around them and not being Mum, not really being Mum. That was hard, you know.’*

(Anna, age 21: sepsis and hysterectomy)

Although most women made a good recovery, it often took weeks or months. Scarring can be a problem for some women who have had life-threatening complications. Both frightening and emotionally upsetting, it could also result in ongoing physical problems. Several women were dismayed as they realised they were going to be less fit for the rest of their lives. One woman developed long-term digestive problems caused by her internal scar tissue:

‘It is a very odd thing, you catch yourself thinking, oh now I’ve become someone who is going to be less well for the rest of my life, and that is a weird feeling.’

(Hannah, 34, uterine rupture)

### Emotional recovery

Finding out what had happened, and coming to terms with the seriousness of their illness, was often emotionally difficult for women. There was great variation in how these traumatic events affected people. Some felt it did not affect their mental health, but others did, and described anxiety, panic attacks, flashbacks, and post-traumatic stress disorder (PTSD) in the aftermath of their experiences. Partners could experience these, as well as the women.

Debbie had a uterine rupture and was advised that she needed to seek psychological help:

‘”Because you’ve gone through trauma”. And she actually said, “If you’re in a car crash nobody expects you just to get up on your feet again and carry on as normal, as soon as you’re physically healed, you know, there’s issues you need to talk about and fears.” And she said, “There’s no difference here with you. You’ve gone something very traumatic, and you should speak to somebody about it, if you’re not quite ready to move on”.’

(Debbie, 29, uterine rupture)

There was variation in when women and men first experienced anxiety, depression, or PTSD, how severe it was, and how long it took to recover. Lisa, 35, was interviewed a year after her daughter was born. She described a very difficult year as she experienced anxiety and panic attacks. But felt she was getting over it and starting to feel a lot better: *‘I want to move on now’*.

Cara, 29, said she was *‘very depressed and manic about my research into what had happened’*, in the first year, but each year got easier. But for some, their child’s first birthday, marking the anniversary of their trauma, stirred emotions:

*‘It was actually a year after that I felt that I needed more help.* [um] *But I think that it just brought feelings out that I’d, I’d just bottled them up. I’d kind of packaged them away and said, that dealt with, and I hadn’t worked through my feelings, I’d just pushed them to one side and said, I don’t, that’s fine, that’s okay.* [um] *Put a smiley face on and got on with it.* [laughs] [um] *And it worked short term, but then it came back to bite me when I was least expecting it as well actually and so I struggled quite a lot around his birthday.’*

(Alison, 30, postpartum haemorrhage and hysterectomy)

Some were offered counselling through the hospital, GP, or health visitor. Some sought counselling themselves. While some did not find it very helpful, many did. Others wanted counselling but were not offered any. There was great variation in when the women felt ready to talk: some quite soon, others not for a few months or even over a year.

### Long-lasting effects

While some women seemed resilient and their emergency experience did not appear to have long-lasting effects on them or their families, others were more affected.

#### Future pregnancy or fertility

Life-threatening emergencies could have a profound impact on a woman’s fertility and future pregnancies. Some women had hysterectomies to save their lives. While some did not feel this was a big issue if their families were complete, for others this was devastating.

Several women need help weighing up the risks of another pregnancy, either with their GP or a consultant. Some women were still potentially able to get pregnant, but were advised against it by doctors because the risks were too high.

Helen had HELLP syndrome with her first baby (HELLP is a complication of pre-eclampsia characterised by haemolysis, elevated liver enzymes, and low platelets):

*‘But the thing that worries me now, is that if we want to have another child,* [um] *they’ve said that the risk of it happening again is 20–30% which is much higher than what I’d like. And that’s the thing that concerns me more now, that I know what it was and what we went through.’*

(Helen, 31, HELLP syndrome)

#### Other family members

In terms of their relationships, many felt their experiences had made them stronger and brought them closer. For others, staying together after such a traumatic event was challenging, and some relationships did not survive the experience. In terms of the wider family, there was a range of views. Some felt their emergency had had a profound effect on their children, while others felt it had little impact or even improved their relationships with them.

### Support

Women’s experience of support varied. Some felt that the support from their local GP and health visitors was excellent, while others would have liked more support after such a traumatic time in hospital.

#### Examples of good care

Several women felt they had very good care and support after discharge. Women appreciated GPs who were in touch and reassuring, and midwives and health visitors who were aware of what they had been through and visited regularly. Knowing that support was available from their GP made a real difference to women:

‘She’s been in constant contact with us. If I do need to have a chat and things I can go and speak to her … I trust her 101%. She’s just there, they’re just available.’

(Naomi, 35, emergency caesarean and Ogilvie syndrome)

Kerry had panic attacks and anxiety after her haemorrhage. Her GP was very patient, offering support and referral to counselling:

‘He was really patient, because I did keep going back. I thought I had illness after illness. The words that he used did calm me down a lot ... Because I was so frightened that I was going to die after that. I always thought I had something life threatening.’

(Kerry, 25, placenta praevia and haemorrhage)

Hanna had been in hospital for several weeks with pre-eclampsia and a haematoma. When discharged, she was frightened and told to contact her GP who was reassuring and helpful, checking her bloods:

*‘* [S/He] *called me at home to say, “Everything is fine. Don’t worry ... it’ll be fine.” So for 2 weeks I was in contact with my GP and they really, really took care of me.’*

(Hanna, 37, pre-eclampsia)

#### Lack of support

However, several women were surprised there was no communication between the hospital and GP, who seemed to have no idea what had happened. Some women felt there was very little support for them after discharge from hospital.

Rob’s wife had placenta praevia and a hysterectomy. He went on to develop PTSD himself. Rob felt their GP was ‘worse than useless’ and as a family they were left with little support. Lack of support may affect how women and their families recover. Sophie and Tom struggled after she had a pulmonary embolism and haemorrhage. They were told there was no help available for her after she was discharged and at home looking after her newborn and toddler:

‘And I couldn’t understand that, because I was still at risk. I still had a pulmonary embolism. And I said, “Well what happens if I’m on my own with the girls and I have a heart attack? Is there anything that we can do to reduce the impact on them if that were to happen?” And they just came up with nothing really.’

(Sophie, 36, pulmonary embolism and haemorrhage)

Some women described putting on a brave face and this may have contributed to them not getting the support that they needed. Joanna said her GP was very supportive but she often put on a front when seeing him, so perhaps he was not aware how much the death of her baby had affected her. Ciara and Michelle were asked to complete the Edinburgh Postnatal Depression Scale with their health visitor, but said it was easy to know what the right answers were so as to appear as if they were fine.

## DISCUSSION

### Summary

This study demonstrates the profound long-term impact a near miss in childbirth can have on new mothers. Their physical recovery could take a long time, and women were often not able to look after their babies as they wished in the early days. Emotional recovery was often difficult, with some women reporting anxiety, panic attacks, flashbacks, and PTSD. Long-lasting effects after discharge from hospital included fears about future fertility and pregnancies, social isolation, and lack of support. Other family members were also potentially affected.

Several women received very good care and support after discharge, demonstrated by GPs being in touch and reassuring, and midwives and health visitors being aware of what had happened and visiting regularly. There is already some follow-up in place in the NHS; currently after discharge most women are visited by a midwife and then a health visitor who usually carry out a postnatal depression screen. However, this study highlights the importance of communication between primary and secondary care, and demonstrates that knowing proactive support was available from their GP team made a real difference to women.

### Strengths and limitations

There are limitations to this study. The data presented are based on interviews with 36 women and 11 partners, and as with qualitative studies aiming for a maximum variation sample, the findings are not intended to be numerically representative. However, the sample was large enough to reach data saturation and included good variation in socioeconomic mix. Participants were mostly of white British origin and despite inclusion of women from Pakistani and Somali backgrounds, the ethnic diversity in the sample is not as wide as it might be. While no account is static — people’s views and interpretation of their experiences are likely to change over time — an effort was made to interview individuals who were both close to events as well as those who were talking about experiences that had happened several years previously.

### Comparison with existing literature

There are relatively few studies worldwide that have reported on the impact of life-threatening conditions in childbirth. The current study’s findings resonate with others reporting emotional trauma and long-lasting effects.^2^,^4^–^6^,^15^ While there is agreement that traumatic events (outside of childbirth) can have significant impact on individuals’, families’, and communities’ abilities to cope, the most effective ways of providing psychological help are not clear.^16^ The literature surrounding PTSD after childbirth is sparse and the authors are not aware of any research on how to support women and their families after a near miss. While studies have highlighted the potential long-term impact of a traumatic birth,^7^ there is little conclusive evidence on the best way to support women and their families. While several studies have suggested the importance of social support and community resources in supporting women after a birth trauma,^17^ the women who have experienced a near miss often appear to be unable to access standard support due to feeling their experiences are so extreme or different to the norm. In addition, systematic reviews have shown that evidence for midwifery-based counselling or formal postnatal debriefing interventions to effectively address mental health problems (including PTSD) is inconclusive.^18^–^20^ However, Rowan *et al*^19^ suggested offering women the opportunity to talk and the importance of health professionals being alert to the signs and symptoms of mental health problems after childbirth.

### Implications for research and practice

There has been little research on the long-term impact of traumatic birth and how best to help women. There is inconclusive evidence on the impact of debriefing programmes. However, we know that those most likely to be well after childbirth are women who had no complications, no worries about their labour and birth, and are given information about their choices for care.^21^

Women who experience a near miss have had none of these. For them, there is often no follow-up from hospital obstetric or midwifery staff. Primary care teams should be made routinely aware if a woman has had a near miss so that they can offer the support these women may need, and be aware that these new mothers may be isolated from their peers and therefore potential support networks. GPs and health visitors should be alert for mental health problems developing, mindful of the impact that the near-miss experience can have on the whole family (including the woman’s partner and other children), and be prepared to offer advice about future pregnancies.
